# Supplementary material for: Changes in Parents’ COVID-19 Vaccine Hesitancy for Children Aged 3–17 Years before and after the Rollout of the National Childhood COVID-19 Vaccination Program in China: Repeated Cross-Sectional Surveys
Source: Vaccines (Basel). 2022 Sep 6;10(9):1478. doi: 10.3390/vaccines10091478 (PMC9502861; doi:10.3390/vaccines10091478)
Supplement: Supplementary file 1 [file vaccines-10-01478-s001.zip › vaccines-1859460-supplementary.pdf]

Questionnaire used in the first round of survey

Part 1:

1-1 Do you have any child?

□1 No □2 Yes, all of them are at least 18 years old

□3 Yes, at least one of them is under the age of 18 years --- Please answer the following questions

1-2 How old is your child who is under the age of 18 years? (If you have more than one child under the age the age of 18 years, please refer to the one whose birthday is closest to today when answering the following questions)

【 】 years

1-3 COVID-19 vaccines developed by China are likely to become available by the end of 2020. What is your likelihood of having your child under the age of 18 years take up free COVID-19 vaccination provided by the government?

□1 Very unlikely □2 Unlikely □3 Neutral □4 Likely □5 Very likely

Part 2:

2-1 Do you agree with the following statements related to COVID-19 vaccination? There is no right or wrong answers.

	**Disagree**	**Neutral**	**Agree**
A. COVID-19 vaccination is highly effective in protecting your child from COVID-19	1	2	3
B. Taking up COVID-19 vaccination can contribute to the control of COVID-19 in China	1	2	3
C. China will have adequate supply of COVID-19 vaccination	1	2	3
D. Your child will have severe side effects after receiving COVID-19 vaccination	1	2	3
E. The protection of COVID-19 vaccines will only last for a short time	1	2	3
F. Your child is afraid of vaccination	1	2	3
G. You do not have time to take your child for COVID-19 vaccination	1	2	3
H. Your family member would support you in having your child take up COVID-19 vaccination	1	2	3
I. Having the child receive COVID-19 vaccination is easy for you if you want them to	1	2	3

2-2 Frequency of exposing to the following information related to COVID-19 vaccination on social media (WeChat, WeChat moments, Weibo, Tiktok, etc.) in the past month

	**Almost never**	**Seldom**	**Sometimes**	**Always**
A. Experiences related to COVID-19 vaccination shared by recipients	0	1	2	3

2-3 Frequency of facemask wearing in public places/transportations other than workplaces in the past month

□1 Every time □2 Often □3 Sometimes □4 Never

2-4 Frequency of facemask wearing when you have close contact with other people in workplace in the past month

□1 Every time □2 Often □3 Sometimes □4 Never

2-5 Frequency of sanitizing hands (using soaps, liquid soaps or alcohol-based sanitizer) after returning from public spaces or touching public installation

□1 Every time □2 Often □3 Sometimes □4 Never

2-6 In the past month, did you……	**Yes**	**No**
A. Avoid social/meal gathering with other people who do not live together	1	2
B. Avoid crowed places	1	2

Last part:

3-1 How old are you: _____ years

3-2 What is your gender? □1Male □2 Female

3-3 What is your relationship status?

□1 Without a stable boyfriend/girlfriend
□2 With a stable boyfriend/girlfriend
□3 Married
□4 Divorced/widowed

3-4 What is your education level?

□1 Primary school or below
□2 Junior high
□3 Senior high or equivalent
□4 College
□5 University
□6 Postgraduate

3-5 What is your monthly income level?

□1 Below 1000RMB
□2 1000-2999 RMB
□3 3000-4999 RMB
□4 5000-6999 RMB
□5 7000-9999 RMB
□6 10,000 RMB or above
□7 No fixed income

3-8 Are you a frontline worker or a management staff?

□1 Frontline worker □2 Management staff

End of Questionnaire

Questionnaire used in the second round of survey

Part 1:

1-1 Do you have any child?

□1 No □2 Yes, all of them are at least 18 years old

□3 Yes, at least one of them is under the age of 18 years --- Please answer the following questions

1-2 How old is your child who is under the age of 18 years? (If you have more than one child under the age the age of 18 years, please refer to the one whose birthday is closest to today when answering the following questions)

【 】years

1-3 Number of doses of COVID-19 vaccination received by your child

□1 0 --- please answer 1-3A □2 1 □3 2

1-3A What is your likelihood of having your child under the age of 18 years take up free COVID-19 vaccination provided by the government?

□1 Very unlikely □2 Unlikely □3 Neutral □4 Likely □5 Very likely

Part 2:

2-1 Do you agree with the following statements related to COVID-19 vaccination? There is no right or wrong answers.

	Disagree	Neutral	Agree
A. COVID-19 vaccination is highly effective in protecting your child from COVID-19	1	2	3
B. Taking up COVID-19 vaccination can contribute to the control of COVID-19 in China	1	2	3
C. China will have adequate supply of COVID-19 vaccination	1	2	3
D. Your child will have severe side effects after receiving COVID-19 vaccination	1	2	3
E. The protection of COVID-19 vaccines will only last for a short time	1	2	3
F. Your child is afraid of vaccination	1	2	3
G. You do not have time to take your child for COVID-19 vaccination	1	2	3
H. Your family member would support you in having your child take up COVID-19 vaccination	1	2	3
I. Having the child receive COVID-19 vaccination is easy for you if you want them to	1	2	3

2-2 Frequency of exposing to the following information related to COVID-19 vaccination on social media (WeChat, WeChat moments, Weibo, Tiktok, etc.) in the past month

	Almost never	Seldom	Sometimes	Always

A. Experiences related to COVID-19 vaccination shared by recipients	0	1	2	3
B. COVID-19 pandemic is not under control in some countries after scaling up COVID-19 vaccination	0	1	2	3
C. Infectiousness and harms of the variants concern of COVID-19	0	1	2	3
D. Outbreak caused by variants concern of COVID-19 in some places of China	0	1	2	3
E. People contract COVID-19 after receiving primary series of COVID-19	0	1	2	3

2-3 Frequency of facemask wearing in public places/transportations other than workplaces in the past month

□1 Every time □2 Often □3 Sometimes □4 Never

2-4 Frequency of facemask wearing when you have close contact with other people in workplace in the past month

□1 Every time □2 Often □3 Sometimes □4 Never

2-5 Frequency of sanitizing hands (using soaps, liquid soaps or alcohol-based sanitizer) after returning from public spaces or touching public installation

□1 Every time □2 Often □3 Sometimes □4 Never

2-6 In the past month, did you……	**Yes**	**No**
A. Avoid social/meal gathering with other people who do not live together	1	2
B. Avoid crowed places	1	2

Last part:

3-1 How old are you: _____ years

3-2 What is your gender? □1Male □2 Female

3-3 What is your relationship status?

□1 Without a stable boyfriend/girlfriend
□2 With a stable boyfriend/girlfriend
□3 Married
□4 Divorced/widowed

3-4 What is your education level?

□1 Primary school or below
□2 Junior high
□3 Senior high or equivalent
□4 College
□5 University
□6 Postgraduate

3-5 What is your monthly income level?

□1 Below 1000RMB
□2 1000-2999 RMB
□3 3000-4999 RMB
□4 5000-6999 RMB

□5 7000-9999 RMB

□6 10,000 RMB or above

□7 No fixed income

3-8 Are you a frontline worker or a management staff?

□1 Frontline worker □2 Management staff

End of Questionnaire
